# Child and adolescent mental health trajectories in relation to exclusion from school from the Avon Longitudinal Study of Parents and Children

**DOI:** 10.1111/camh.12367

**Published:** 2020-01-21

**Authors:** María Tejerina‐Arreal, Claire Parker, Amelia Paget, William Henley, Stuart Logan, Alan Emond, Tamsin Ford

**Affiliations:** ^1^ College of Medicine and Health University of Exeter Exeter UK; ^2^ Musgrove Park Hospital Taunton UK; ^3^ Bristol Medical School University of Bristol Bristol UK; ^4^ Department of Psychiatry University of Cambridge Cambridge UK

**Keywords:** Avon Longitudinal Study of Parents and Children, school exclusion, child psychopathology, gender differences

## Abstract

**Background:**

As the prevalence of childhood mental health conditions varies by age and gender, we explored whether there were similar variations in the relationship between psychopathology and exclusion from school in a prospective UK population‐based birth cohort.

**Method:**

The Avon Longitudinal Study of Parents and Children collected reports of exclusion at 8 years and 16 years. Mental health was assessed at repeated time points using the Strengths and Difficulties Questionnaire (SDQ).

**Results:**

Using adjusted linear mixed effects models, we detected a nonlinear interaction between exclusion and age related to poor mental health for boys [adjusted coefficient 1.13 (95% confidence interval 0.55–1.71)] excluded by age 8, but not for girls. The SDQ scores of boys who were excluded in primary school were higher than their peers from age 3, and increasingly diverged over time. As teenagers, these interactions appeared for both genders [boys’ adjusted coefficient 0.18 (0.10–0.27); girls 0.29 (0.17–0.40)]. For teenage girls, exclusion by 16 was followed by deteriorating mental health. Family adversity predicted exclusion in all analyses.

**Conclusion:**

Prompt access to effective intervention for children in poor mental health may improve both mental health and access to education.

**Key Practitioner Message:**

- Children who were subsequently excluded from school often faced family adversity and had poor mental health, which suggests the need for an interdisciplinary response and a multiagency approach.
- Poor mental health may contribute to and result from exclusion from school, so both mental health and education practitioners have a key role to play.
- Boys who enter school with poor mental health are at high risk of exclusion in primary school, which prompt assessment and intervention may prevent.
- Both boys and girls who are excluded between the ages of 15 and 16 years may have poor, and in the case of girls, deteriorating, mental health.

## Abstract 2

Read the commentary on this article at 10.1111/camh.12395

## Introduction

The role of schools in children's mental health is a current policy focus in the U.K. (Department of Health & Social Care & Department for Education, 2018). Excluded pupils are more likely to suffer long‐term mental health problems, although there are heated debates about the direction of causality (Gill, 2017; Hemphill et al., 2010). Prospectively collected data at population level offer an opportunity to study temporal relationships between poor mental health and exclusion from school, but given that the latter is uncommon, sufficiently large data sets are rare. Several studies suggested a strong relationship between psychiatric disorder and exclusion from school (Bowman‐Perrott et al., 2011; Ford et al., 2012; Paget et al., 2018; Parker et al., 2016, 2018; Patton et al., 2014; Whear et al., 2013). Analyses of data from a nationally representative population sample suggested a bidirectional relationship between exclusion and poor mental health (Ford et al., 2018), indicating that effective intervention to support the learning and mental health of children who struggle with school could prevent both future mental disorders and exclusions (Beckett et al., 2010).

Children excluded from school often face multiple vulnerabilities; analysis of the Avon Longitudinal Study of Parents and Children (ALSPAC) demonstrated that exclusion was more likely among children of lower socioeconomic status, boys and those with language difficulties, lower educational attainment or special educational needs (SEN; Paget et al., 2018). Family characteristics, such as poor parental mental health and engagement with education, also predicted exclusion. Children who were subsequently excluded were more likely to have a psychiatric disorder, behavioural difficulties and social communication problems, as well as involvement in bullying as a perpetrator or victim and poor teacher‐pupil relationships.

The onset and pattern of mental health conditions vary by both age and gender (Cohen et al., 1993; Merikangas et al., 2010; Wesselhoeft et al., 2015). Boys are more likely to develop attention deficit hyperactivity disorder (ADHD) and autistic spectrum conditions (ASC), which mostly present in childhood, as well as conduct and oppositional disorders (Jensen & Steinhausen, 2015; Zahn‐Waxler, Shirtcliff & Marceau, 2008). These externalising disorders often impair children's ability to cope with school and predicted exclusion in the three‐year follow‐up of the 2004 British Child and Adolescent Mental Health Survey (Parker et al., 2018). Furthermore, emotional disorders display a male preponderance before the age of 12 years and a female preponderance thereafter (Rutter, Caspi, & Moffitt, 2003; Sadler et al., 2018; Wesselhoeft et al., 2015; Zahn‐Waxler et al, 2008).

Improved understanding of the mental health trajectories of children who are subsequently excluded could assist education and mental health practitioners to better target assessment and interventions. The examination of mental health trajectories related to exclusion is important to disentangle the influence of mental health and exclusion on each other. The longitudinal perspective of the ALSPAC data source offers an excellent opportunity compared with retrospective or cross‐sectional data to study these trajectories. We aimed to undertake an exploratory analysis of mental health before and after exclusion from school in a prospectively studied birth cohort. Given the gender and age differences in the pattern of mental health conditions and exclusion from school, we studied trajectories separately for boys and girls, separated into primary and secondary school ages.

## Methods

Ethical approval for the study was obtained from the ALSPAC Ethics and Law Committee and the Local Research Ethics Committees as the Peninsula College of Medicine and Dentistry Research Ethics Committees (11/09/120).

### Sample

Avon Longitudinal Study of Parents and Children is an ongoing longitudinal birth cohort study that recruited 14,541 pregnant women resident in Avon, South West England, between April 1991 and December 1992, with 13,988 alive at 1 year old (Boyd et al., 2013). The sample and phases of enrolment are described in the cohort profile papers (Boyd et al., 2013; Fraser, et al., 2013). The study website contains details of all the data that are available through a fully searchable data dictionary and variable search tool http://www.bristol.ac.uk/alspac/researchers/ourdata/. Version number for the data used is B1119.

This analysis is based on 8248 children with data on exclusion at 8 years (56% of the total cohort alive at 1 year) and 4482 with data on exclusion at 16 years.

### Measures

#### Mental health

Mental health was measured using the Strengths and Difficulties Questionnaire (SDQ, Goodman, 2001), a validated questionnaire for common childhood psychopathology with parallel versions for parents, teachers and children aged 11 years or more (Cronbach alpha .73, test–retest reliability of .62). It comprises 25 statements that contribute to five subscales, which measure emotional symptoms, conduct problems, hyperactivity/inattention, peer problems and prosocial behaviour. A total difficulties score is derived by summing the subtotals of the first four subscales and ranges from 0 to 40; higher score indicates greater distress. The SDQ was completed by parents at 3, 6, 8, 9, 11, 13 and 16 years old.

#### Exclusion from school

##### Exclusion by 8 years

When the child was 8 years 7 months old, mothers were asked if their child had ever been excluded from school, creating a binary variable of ‘Yes’ or ‘No’ (*n* = 53, *n* = 8195, respectively). All others were coded as missing data, which included those who did not complete the question (*n* = 6443).

##### Exclusion at 16 years

At 16 years old, mothers and young people were asked separately about fixed term and permanent exclusions from school in the past 12 months. An overall binary variable was derived: coded as ‘Yes’ if either the young person or parent reported fixed term or permanent exclusion (*n* = 391), or ‘No’ if both reported no exclusions (*n* = 4091). Questionnaires not sent or completed were coded as missing (*n* = 10,209).

#### Confounders and adjusting the models

The Family Adversity Index (FAI) focused on the first 2 years of the index child's life and used in this study to control for the impact of multiple confounding factors (Steer et al., 2004). It included 18 items about age of the mother at pregnancy, housing, mother's education, financial difficulties, partner relationship status, support and cruelty, family size and care, social network, parent affective disorder, drug's addiction and crime. Total FAI score was gained by summing all 18 items, with higher scores indicating greater family adversity.

### Statistical analysis

Analyses were conducted using Stata version 15.0 (StataCorp, 2017) and were completed separately for boys and girls in relation to exclusion by age 8 and age 16. Linear mixed effect models for SDQ total difficulties score trajectories were executed and adjusted for FAI, SEN and ethnicity as potential confounders (Paget et al., 2018). Using backwards stepwise regression, based on coefficient size and standard errors, variables were removed to build the final models. All analyses (Stata do‐files) are available at https://github.com/MariaTejerina-Arreal/trajectories/ https://doi.org/10.5281/zenodo.3531590.

Mental health (SDQ) parent's report at the seven age points mentioned above accounted in the models and was tested in relation to exclusion at 8 and 16 years.

**Figure 1:**
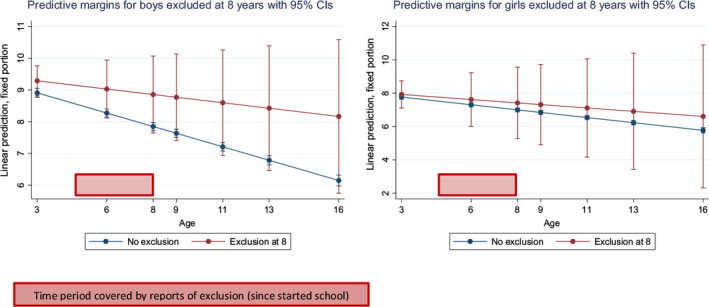
Trajectories (3−16 years) of parent‐reported SDQ total difficulties score for boys and girls excluded or not from school by 8 years

**Figure 2:**
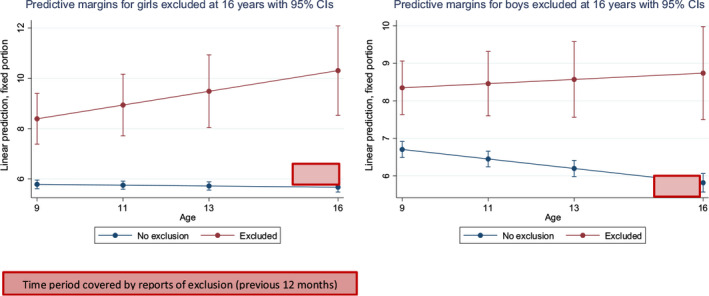
Trajectories (9−16 years) of parent‐reported SDQ total difficulties score for boys and girls excluded or not from school by 16 years

Linear predictions from the models with 95% confidence intervals were plotted to visualise these trajectories, in which the time period covered by reports of exclusions is specified (red boxes in Figures 1 and 2). Predictive margins assess the predicted level of SDQ total difficulties score based on change on age and the difference in margins between those with and without reports of exclusion from school.

## Results

A full description of the characteristics of children who were and were not excluded is available from Paget et al. (2018). Main factors associated with exclusion at both time points included male gender, lower socioeconomic status, maternal psychopathology, social communication difficulties, language difficulties, antisocial activities, bullying/being bullied, lower parental engagement with education, poor relationship with teacher, low educational attainment and special educational needs.

Reassuringly few children (*n* = 53/8195) were excluded by 8 years of age; of these, only 13 were girls. At 16 years, 391 young people were excluded from school (391/4091): 145 girls and 246 boys. The remaining 3731 children had no reported exclusion, while 9 had reported exclusions at both 8 and 16 years and were included in both analyses.

### Mental Health (SDQ) trajectories between 3 and 16 years in boys and girls excluded or not excluded from school by aged 8

There were strong unadjusted associations between exclusion from school by 8 years and SDQ total difficulties score between the age of 3–16 years [coefficient 4.91 (CI: 3.80–6.02) *p* < .001)]. This trend continued when the analysis focused only on children aged 9–16 years who had been excluded from school by 8 years compared to children who had not been excluded [coefficient 5.23 (3.99‐6.46) *p* < .001].

There was no statistically significant difference in the levels of psychopathology in girls between those who had or had not been excluded by 8 years, while boys who had been excluded had significantly higher levels of psychopathology from age three, with a peak in the latter years of primary school. (See Figure 1 and Table 1) increasingly diverged from their peers over time. There was a significant nonlinear interaction between age in relation to mental health and exclusion at 8 years old for boys, but not for girls (Table 1). The FAI predicted poor mental health for boys and girls excluded from school by age 8.

**Table 1:** Parent‐reported SDQ total difficulties trajectories between 3 and 16 years in boys and girls who were or were not excluded from school by 8 years of age

Exclusion at 8 years *N* with data 8248				
	Girls	*N*	Boys	*N*
		4136 No		4159 No
		13 Yes		40 Yes

SDQ	Adjusted Coef (95% CI)	*p*‐Value	Adjusted Coef (95% CI)	*p*‐Value
Age	−.53 (−0.59 to −0.48)	<.001	−.34 (−0.40 to 0.29)	<.001
Age squared	.02 (0.018 to 0.023)	<.001	.00 (0.00 to 0.01)	<.001
Exclusion	.74 (−3.5 to 4.9)	.733	.34 (−2.08 to 2.78)	.780
Exclusion × age interaction	−.18 (−1.1 to 0.8)	.715	1.13 (0.55 to 1.71)	<.001
Exclusion × age squared interaction	.01 (−0.04 to −0.07)	.630	−.06 (−0.08 to −0.03)	<.001
FAI (Family Adversity Index)	.61 (0.55 to 0.67)	<.001	.60 (0.55 to 0.67)	<.001

Special educational needs was not associated in any of the models, while ethnicity was only associated for boys excluded at 8 years. We also explored quadratic age exclusion interactions in all the models, but this was only significant for boys excluded at 8 years (Table 1).

### Mental health (SDQ) trajectories between 9 and 16 years in boys and girls excluded versus not excluded from school between the age of 15 and 16 years

Unadjusted analysis revealed higher mean parent‐reported SDQ total difficulties score from 9 to 16 years for young people who had been excluded from school at 16 compared with children who had not been excluded at this time point [coefficient 2.95 (2.52–3.38) *p* < .001].

Although more children had experienced exclusion at 16, we found modest significant interactions between exclusion and age on mental health in the SDQ‐adjusted models (Table 2) where exclusion was not significant in the prediction of psychopathology.

**Table 2:** Parent‐reported SDQ total difficulties trajectories between 9 and 16 years in boys and girls who were or were not excluded from school by between the age of 15 and 16 years

Exclusion at 16 years *N* with data 4482				
	Girls	*N*	Boys	*N*
		2324 No		1767 No
		145 Yes		246 Yes

SDQ	Adjusted Coef (95% CI)	*p*‐Value	Adjusted Coef (95% CI)	*p*‐Value
Age	−.02 (−0.04 to 0.00)	.187	−.12 (−0.15 to −0.10)	<.001
Exclusion	−1.24 (−2.77 to 0.29)	.111	.28 (−0.89 to 1.4)	.641
Exclusion × age interaction	.29 (0.17 to 0.40)	<.001	.18 (0.10 to 0.27)	<.001
FAI (Family Adversity Index)	.58 (50 to 0.66)	<.001	.57 (46 to 0.68)	<.001

The mental health of girls excluded from school at 16 years appeared to be on a deteriorating trajectory compared with their peers not excluded. In contrast, mental health remained fairly stable for boys, although poorer compared to their peers not excluded (Figure 2).

FAI predicted exclusion between 15 and 16 years for both genders.

Analyses on which selected models were based are available at https://github.com/MariaTejerina-Arreal/trajectories/ https://doi.org/10.5281/zenodo.3531590.

## Discussion

We found age‐ and gender‐specific differences in mental health trajectories in relation to school exclusion, with clear evidence of poor mental health prior to and after exclusion. For teenage girls, there was tentative evidence that their mental health continued to deteriorate following exclusion, consistent with findings that exclusion from school has a bidirectional relationship with childhood psychiatric disorder in a population sample of British school children followed for 3 years (Ford et al., 2018). This is particularly concerning given the high prevalence of mental health conditions detected in teenage girls (Sadler et al., 2018) and recent reported increases in fixed term and permanent exclusions per school enrolment (Department for Education, 2018b).

It is also important to emphasise the strong and universal prediction of poor mental health by early family adversity, which echoes strong relationship between lower socioeconomic status and exclusion (Gill, 2017; Paget et al., 2018). Policies to support young families from deprived areas might therefore improve educational as well as health outcomes (Department of Education, 2016; Ryder, Edwards & Rix, 2017).

These results confirm well‐established age and gender differences in childhood psychopathology (Sadler et al., 2018). Boys are more likely to experience disruptive behavioural and neurodevelopmental problems, most common in early childhood, which predictably interfere with early academic attainment and later educational outcomes (Wesselhoeft et al., 2015; Zbar, Surkan, Fombonne, & Melchior, 2016). Children with behavioural problems often have cognitive difficulties from preschool onwards and hence may struggle to learn to adapt their behaviour to the classroom context and experience other learning problems (Althoff et al., 2010; Galéra et al., 2012). Boys who were excluded from the early years of primary school started their school careers with significantly poorer mental health than their peers who were not subsequently excluded. The lack of similar differences in trajectory for girls at primary school was almost certainly explained by the extremely low number of girls who experienced exclusion by age 8 and the resulting lack of power for analysis. While there was no statistically significant relationship (see Figure 1), girls excluded by age 8 also had consistently elevated levels of poor mental health.

While the number of exclusions increased in both genders between the ages of 8 and 16 years, the increase for girls was particularly notable. Girls are at higher risk of emotional disorders and difficulties in late childhood and adolescence (Cohen et al., 1993; Sadler et al., 2018; Wesselhoeft et al., 2015) and tend to experience a longer duration of emotional problems (Essau et al., 2010). Emotional disorders are less likely to be recognised by parents or teachers, and unrecognised psychiatric disorder also carries an increased risk of exclusion from school (Department of Health & NHS England, 2014; Parker et al., 2018). Emotional disorders can influence attendance at school and academic attainment (Finning et al., 2019; Riglin, Petrides, Frederickson, & Rice, 2014), while associated irritability, apathy and poor concentration may provoke disciplinary problems. Previous research (Zbar et al., 2016) suggests that children with emotional problems were nearly twice as likely to experience academic failure by adolescence than their healthier peers. Finally, exclusion from school, particularly if permanent, disrupts peer relationships and self‐image and may increase social isolation, all of which may predispose to the development of anxiety or depression.

Our findings emphasise the complex inter‐relationship between poor mental health and exclusion from school. Children educated in alternative provision are less likely to achieve good grades (Department of Education, 2018a), and more likely to become NEET (Not in Education, Employment or Training) after the age of 16 and in contact with the criminal justice system (Department of Education, 2018b). However, properly resourced alternative provision offers the opportunity of transformative education, but requires adequately trained and supported teachers for children facing trauma and mental health problems alongside learning needs (Gill, 2017).

It is important to avoid therapeutic nihilism. Early and effective assessment and remediation of both mental health and educational needs may improve outcomes in both domains. There are effective evidence‐based interventions for many mental health conditions (Weisz et al., 2017); sadly, however, only a small proportion of children with clinically significant mental health conditions access mental health services (Ford, Hamilton, Meltzer & Goodman, 2007; Mandalia, et al., 2018). Given the deteriorating trajectory, our findings suggest these children should be our first priority for intervention.

Disruptive behaviour is particularly difficult to manage within the school. School‐based responses are important and effective for school‐based problems (Axford et al., 2019; Weisz et al., 2017). Given that conduct disorder is one of the commonest childhood mental health conditions (Sadler et al., 2018) and predicts to all adult mental health conditions (Kim‐Cohen et al, 2003), we should be ensuring teachers are adequately trained in classroom management and that parents who are concerned about their child's behaviour have easy access to parent training courses.

A developing evidence‐base suggests that training teachers to manage behaviour using strategies that incorporate positive reinforcement reduces behavioural problems and improves mental health among primary school pupils (Moore et al., 2019; Nye, Melendez‐Torres & Gardner, 2018). Individualised approaches seem most effective for children with severely challenging behaviour, but there is insufficient research into behavioural management in secondary schools (Moore et al., 2019). A recent multimethod systematic review of school‐based psychosocial interventions for children with ADHD demonstrated small to medium effect sizes for both educational attainment and ADHD symptoms, in addition to suggesting that individually tailored programmes and those that focus on improving emotional regulation were the most effective (Moore et al., 2019). Similarly, another systematic review suggests that school‐based programmes can reduce exclusions (Valdebenito et al., 2018).

The additional demands placed on schools' staff time and resources are huge (Newlove‐Delgado et al., 2015; Snell et al., 2013). There are few economic evaluations of school‐based interventions, but training a teacher potentially benefits all the children that they subsequently teach and will likely be significantly more cost‐effective than intervening directly with successive cohorts of children. Estimates for each educational lifetime cohort of permanently excluded pupils run to £2.1 billion for every year's excluded cohort to the education, health, benefits and criminal justice sectors. Yet, as the full extent of exclusion greatly exceeds official figures, the true cost is likely to be many multiples of this estimate (Gill, 2017). Encouragingly, worldwide there is an increasing focus on school‐based mental health services and interventions (Murphy et al., 2017). Laying aside the individual costs of poor mental health and exclusion from school, if such services and interventions were adequately resourced and implemented, they may save considerable sums from health, education and criminal justice budgets.

## Strengths and limitations

Population‐based quantitative research on exclusion from school in relation to mental health remains relatively rare, as there are few suitable data sets. This analysis benefited from a large population‐based birth cohort that was studied over an extended period, as well as validated measures.

However, there are methodological considerations. As with all secondary analyses, we were restricted by the available data. We were unable to differentiate between fixed term (suspension) and permanent (expulsion) exclusions, but would have almost certainly lacked statistical power to explore trajectories for permanent exclusion. In addition, exclusion data were only collected at two time points, and the questions at each time point differed. The lack of data on exclusions between the ages of 8 and 14 years is likely to explain the low number of pupils with exclusion reported at both time points. Despite these limitations, ALSPAC is one of the best data sources available on this vulnerable population.

Attrition from ALSPAC was substantial and systematic; children who dropped out were more likely to suffer from disruptive behaviour disorder and experience family adversity; inevitably, we lacked data on some children from the original cohort who experienced exclusion. This would lead to underestimation of the prevalence of exclusion and reduce statistical power to detect associations. Previous research on the impact of attrition in ALSPAC in relation to behavioural difficulties (Wolke et al., 2009) suggests that our conclusions would be unlikely to change even if complete data were available. The data from ALSPAC relate to young people who attended school between the mid‐1990s and early 21st century, so may not generalise to current influences on mental health and function at school. All of the participating young people were born in one particular region of the UK, as thus findings may not generalise to substantially different populations in the UK and elsewhere.

Finally, we were reliant on parental and young person's report about exclusion; while recall is thought to be good for important and rare events, social desirability may influence informants’ willingness to report them (Althubaiti, 2016). We lacked data from administrative records to triangulate their responses against. These data should be made more easily accessible to researchers in future data collection. Administrative records have their own problems of accuracy; there is a growing concern about schools removing students from their register in ways which would not show up in official exclusion statistics (Children's Commissioner, 2019). The number of children educated in the alternative provision is suspected to greatly exceed the officially reported number of permanent exclusions (Gill, 2017).

## Conclusions

Our findings provide further evidence of the association between mental health and exclusion from school and emphasise the need for a multidisciplinary response to support these highly vulnerable young people. Exclusion from school goes beyond education. Health and mental health professionals have a key role in supporting pupils' who struggle with school and preventing the prospective societal and individual burden associated with exclusion.

## Ethical approval

Ethical approval for the study was obtained from the ALSPAC Ethics and Law Committee and the Local Research Ethics Committees as the Peninsula College of Medicine and Dentistry Research Ethics Committees (11/09/120).
